# Magnetocaloric study, critical behavior and spontaneous magnetization estimation in La_0.6_Ca_0.3_Sr_0.1_MnO_3_ perovskite

**DOI:** 10.1039/c8ra00001h

**Published:** 2018-03-06

**Authors:** M. Jeddi, H. Gharsallah, M. Bejar, M. Bekri, E. Dhahri, E. K. Hlil

**Affiliations:** Laboratoire de Physique Appliquée, Faculté des Sciences, Université de Sfax B. P. 1171 3000 Sfax Tunisia marwajeddi@gmail.com; Institut Préparatoire aux Études d'Ingénieur de Sfax, Université de Sfax B. P. 1172 3018 Sfax Tunisia; Physics Department, Rabigh College of Science and Art, King Abdulaziz University P.O. Box 344 Rabigh 21911 Saudi Arabia; Institut Néel, CNRS Université J. Fourier B. P. 166 38042 Grenoble France

## Abstract

A detailed study of structural, magnetic and magnetocaloric properties of the polycrystalline manganite La_0.6_Ca_0.3_Sr_0.1_MnO_3_ is presented. The Rietveld refinement of X-ray diffraction pattern reveals that our sample is indexed in the orthorhombic structure with *Pbnm* space group. Magnetic measurements display a second order paramagnetic (PM)/ferromagnetic (FM) phase transition at Curie temperature *T*_c_ = 304 K. The magnetic entropy change (Δ*S*_M_) is calculated using two different methods: Maxwell relations and Landau theory. An acceptable agreement between both data is noted, indicating the importance of magnetoelastic coupling and electron interaction in magnetocaloric effect (MCE) properties of La_0.6_Ca_0.3_Sr_0.1_MnO_3_. The maximum magnetic entropy change (−Δ*S*^max^_M_) and the relative cooling power (RCP) are found to be respectively 5.26 J kg^−1^ K^−1^ and 262.53 J kg^−1^ for *μ*_0_*H* = 5 T, making of this material a promising candidate for magnetic refrigeration application. The magnetic entropy curves are found to follow the universal law, confirming the existence of a second order PM/FM phase transition at *T*_c_ which is in excellent agreement with that already deduced from Banerjee criterion. The critical exponents are extracted from the field dependence of the magnetic entropy change. Their values are close to the 3D-Ising class. Scaling laws are obeyed, implying their reliability. The spontaneous magnetization values determined using the magnetic entropy change (Δ*S*_M_*vs. M*^2^) are in good agreement with those obtained from the classical extrapolation of Arrott curves (*μ*_0_*H*/*M vs. M*^2^). The magnetic entropy change can be effectively used in studying the critical behavior and the spontaneous magnetization in manganites system.

## Abstract 2

A detailed investigation was conducted on the magnetocaloric properties of La_0.6_Ca_0.3_Sr_0.1_MnO_3_ and its potential application in cooling fields.

## Graphical abstract

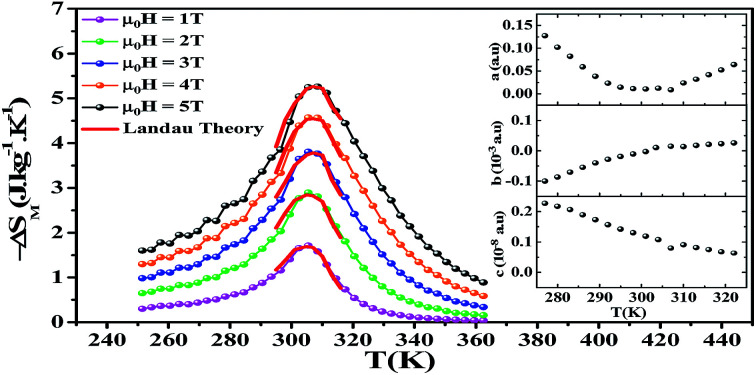

## 1. Introduction

In the last few decades, the study of the Magnetocaloric Effect (MCE) has attracted the attention and whetted the interest of scientific and engineering communities, not only for its potential applications near room temperature but also for other energy conversion matters^1^ as well as certain environmental protection issues. The MCE can be defined as an intrinsic property of magnetic materials. It is characterized by the temperature change (Δ*T*_ad_) in an adiabatic process and by the entropy change (Δ*S*_iso_) in an isothermal process originating uniquely from the application and removal of an external magnetic field in the presence of such ferromagnetic materials as gadolinium which was firstly proposed by G. V. Brown in 1976.^2^

The building of a magnetic refrigeration device near room temperature based on the MCE provides tremendous economic, ecological and energetic benefits compared to the rest of existing refrigeration machines which are based on conventional gas compression/expansion technique.^3–5^ Indeed, since the driving force of magnetic refrigerators arises from the variation of the applied magnetic field, the number of energy consuming elements involved in the refrigeration process is drastically reduced resulting in an enhancement of the cooling efficiency. Moreover, these devices are very environmentally friendly. They do not use any toxic gaseous substances which are normally responsible for damaging our living environment.^6^ It is worthy highlighting that Brown's idea has opened the door to a completely innovative technology which is now under development with a notably huge amount of working prototypes.^7^ The research on magnetocaloric materials presenting optimal magnetocaloric properties^8–10^ was obviously taken further towards the end of the 90's when giant MCE was discovered in Gd_5_Si_2_Ge_2_.^11^ Immediately, hundreds of other materials with extraordinary MCE were found^12–15^ and still today dozens of new materials with giant MCE are described every year. Consequently, several magnetic materials which belong to various chemical families have been fully characterized^16^ with deeper investigation on the most intimate details of the structural and magnetic properties. Recently, large values of MCE have been observed in the perovskite based-manganite of (R_1 − *x*_M_*x*_)MnO_3_ formula (where R is a trivalent rare earth ion and M is a divalent alkali earth ion).^17,18^ With small thermal and magnetic hysteresis, large magnetic entropy change, and relatively low cost,^19^ perovskite manganese oxides have been the subject of continuous research for many years as advantageous materials for refrigeration. This interest arises not only from its dynamic ability for uses in device applications^20–22^ but also from its impressive physical properties.^23–27^ There are numerous sound arguments confirming the fact that perovskite based-manganite compounds will perform a crucial role in the incoming technologies of the near future.^28^

Owing to the large amount of known magnetocaloric materials, it was necessary to develop strategies which enable us to compare them accurately, apart from their nature, processing or composition. Nowadays, significant advances have been carried out allowing a deeper insight to better explore the matter. Phenomenological theories are the key tools which allow us to interpret the performing properties of different magnetocaloric materials. The Landau theory was used to evaluate the importance of magnetoelastic coupling and electron interaction in the magnetocaloric effect.^29,30^ The Mean-field theory was created to establish direct relations between magnetic entropy change and magnetization.^31–33^ The theory of critical phenomena was exploited to justify the existence of a universal magnetocaloric behavior in second-order magnetic phase transition materials.^34,35^

In the present work, a detailed investigation was conducted on magnetocaloric properties of La_0.6_Ca_0.3_Sr_0.1_MnO_3_ compound and its potential application in the cooling fields. Landau mean-field analysis was performed to estimate the magnetic entropy change (Δ*S*_M_) near the Curie temperature. Results are then compared to those obtained using the classical Maxwell relation. A phenomenological universal curve was used as a simple method for extrapolating the magnetic entropy change to confirm the order of the magnetic transition. From the field dependence of isothermal entropy change data, critical exponents were calculated and then verified by the scaling law. From the magnetic entropy change (Δ*S*_M_*vs. M*^2^), spontaneous magnetization (*M*_spont_) was estimated and then compared to that estimated from the classical extrapolation of the Arrott curves (*μ*_0_*H*/*M vs. M*^2^).

## 2. Experiment

### 2.1. Synthesis

The polycrystalline sample of La_0.6_Ca_0.3_Sr_0.1_MnO_3_ was obtained by the mixture of two citric-gel manganite-based oxides; La_0.6_Ca_0.4_MnO_3_ and La_0.6_Sr_0.4_MnO_3_, with mole fractions of 0.75 and 0.25, respectively. Then, the mixed powder 0.75La_0.6_Ca_0.4_MnO_3_/0.25La_0.6_Sr_0.4_MnO_3_ was sintered at 1300 °C to obtain the desired manganite.

The La_0.6_Ca_0.4_MnO_3_ and La_0.6_Sr_0.4_MnO_3_ samples were prepared by citric-gel method^36,37^ using nitrate reagents: La(NO_3_)·6H_2_O, Ca(NO_3_)_2_·4H_2_O, Mn(NO_3_)_2_·6H_2_O and Sr(NO_3_)_2_. The precursors were dissolved in distilled water. Citric acid and ethylene glycol were added to prepare a transparent stable solution. The solution was heated at 80 °C in order to eliminate water excess and to obtain a viscous glassy gel. The solution on further heating at 120 °C led to the emergence of dark grayish flakes which were calcined at 700 °C for 12 h. Then, the powder was pressed into pellets and finally sintered at 900 °C for 18 h.

### 2.2. Characterization

The structure and phase purity of La_0.6_Ca_0.3_Sr_0.1_MnO_3_ were examined by powder X-ray diffraction technique with CuKα radiation (*λ* = 1.5406 Å), at room temperature, by a step scanning of 0.015° in the range of 20° ≤ 2*θ* ≤ 80°. The morphology of the surface was observed by the scanning electron microscopy (SEM). This technique was employed also to prepare a histogram of particle size. The elemental composition of the prepared specimen was checked by the energy dispersive X-ray analysis (EDAX). The magnetization curve *versus* temperature was obtained under an applied magnetic field of 0.05 T with a temperature ranging from 5 to 400 K. Isothermal magnetization data as a function of magnetic field was performed with dc magnetic fields from 0 to 5 T.

## 3. Results and discussion

### 3.1. Structural study

**Fig. 1:**
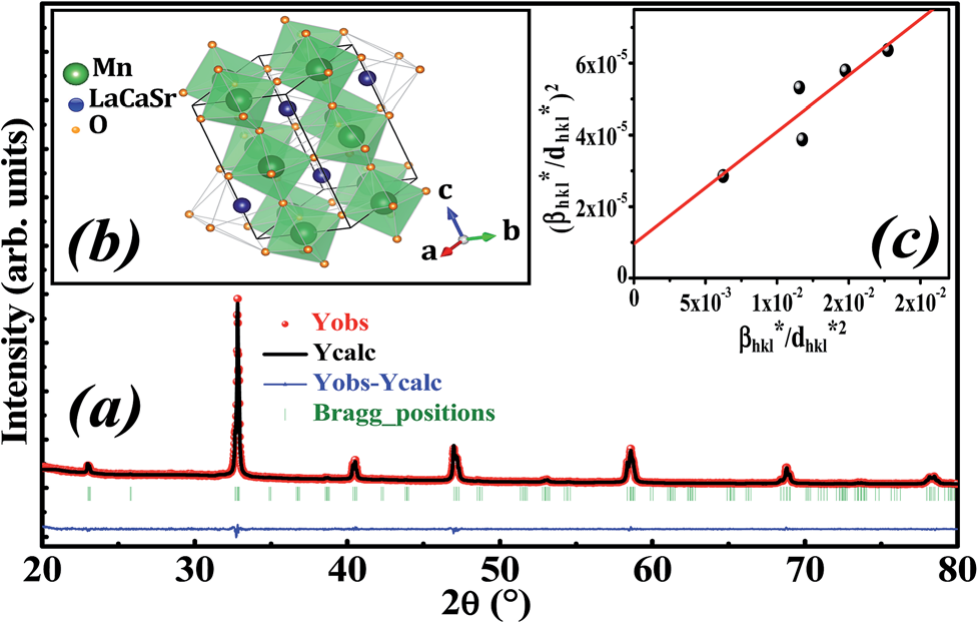
(a) Rietveld refinement, (b) crystal structure and (c) Halder–Wagner plot of La_0.6_Ca_0.3_Sr_0.1_MnO_3_ compound.

Fig. 1a illustrates the X-ray diffraction (XRD) pattern of La_0.6_Ca_0.3_Sr_0.1_MnO_3_ sample. Rietveld refinement is performed by using Fullprof program.^38^ The fitting between the observed and the calculated diffraction profiles shows an excellent agreement, taking into consideration the low value of the fit indicator (*χ*^2^ = 1.897). We notice that the sample is of a single phase without any trace of foreign impurity confirming the high purity of the product material. All the diffraction peaks are indexed in the orthorhombic structure with *Pbnm* space group. The crystal structure of La_0.6_Ca_0.3_Sr_0.1_MnO_3_ is schematically depicted in Fig. 1b. The refinement results are gathered in Table 1.

**Table 1:** Refined structural parameters for La_0.6_Ca_0.3_Sr_0.1_MnO_3_ compound

Compound	La_0.6_Ca_0.3_Sr_0.1_MnO_3_
Space group	*Pbnm*, *a* ≠ *b* ≠ *c*, *α* = *β* = *γ* = 90°
**Cell parameters**	
*a* (Å)	5.48045 (18)
*b* (Å)	5.45131 (18)
*c* (Å)	7.69547 (2)
*V*/FU (Å^3^)	57.477

**Atoms**	
La, Ca, Sr site (*x*, *y*, *z*)	0.9968 (11)
	0.0117 (8)
	0.25000
Mn site (*x*, *y*, *z*)	0.50000
	0.00000
	0.00000
O_1_ site (*x*, *y*, *z*)	0.0562 (4)
	0.4917 (6)
	0.25000
O_2_ site (*x*, *y*, *z*)	0.7174 (3)
	0.2763 (5)
	0.0342 (18)

**Bond angles and bond lengths**	
〈*θ*_Mn–O–Mn_〉 (°)	160.595
〈*d*_Mn–O_〉(Å)	1.959

**Agreement factors**	
*R*_F_ (%)	3.05
*R*_B_ (%)	2.08
*R*_p_ (%)	14.6
*R*_wp_ (%)	10.5
*R*_exp_ (%)	9.05
*χ* ^2^ (%)	1.897

Goldschmidt's tolerance factor *t*_G_ as a criterion for the formation of a perovskite structure is calculated using the following expression:^39^1
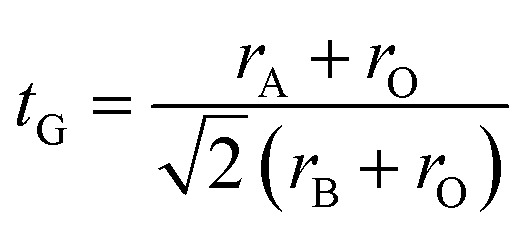
where *r*_A_, *r*_B_ and *r*_O_ are the radii of A, B and O site ions in ABO_3_ structure, respectively.

Oxide-based manganite compounds have a perovskite structure if their tolerance factor is between 0.78 and 1.05.^40^ In the present study, the obtained tolerance factor of La_0.6_Ca_0.3_Sr_0.1_MnO_3_ is 0.925 which is within the stable range of the perovskite structure.

The average crystallite size is obtained from the XRD peaks using the Debye–Scherrer formula:^41^2
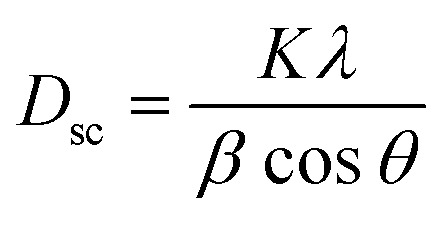
where *λ* = 1.5406 is the wavelength of CuKα radiation, *K* = 0.9 is the shape factor, *β* is the full-width at half-maximum of an XRD peak in radians and *θ* is the Bragg angle.

The mean value of the crystallite size of La_0.6_Ca_0.3_Sr_0.1_MnO_3_ corresponds to 30 nm which confirms the nanometric size of our compound.

The Halder–Wagner (H–W) method is another method to determine the crystallite size. It is expressed as follows:^42^3
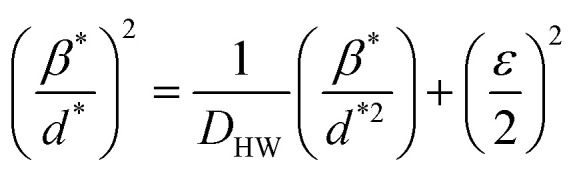
where 
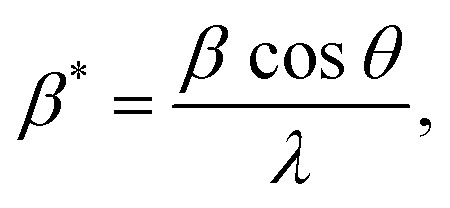

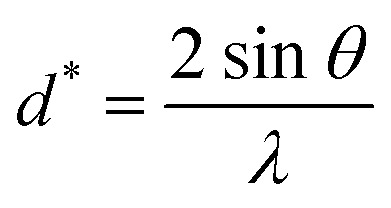
 and *ε* is a coefficient related to strain effect on the crystallites.

The plot of (β^*^/d^*^)^2^ (axis-*y*) as a function of (β^*^/d^*2^) (axis-*x*) corresponding to the 5 strongest peaks of La_0.6_Ca_0.3_Sr_0.1_MnO_3_ is shown in Fig. 1c. The crystallite size *D*_HW_ is achieved from the slope inverse of the linearly fitted data and the root of the *y*-intercept gives the microstrain *ε*. The values of *D*_HW_ and *ε* are found to be respectively 31.9 nm and 0.0062. It is worth noting that the crystallite size calculated by H–W method is slightly higher than that calculated by Debye–Scherrer method because the broadening effect due to the microstrain is completely excluded in Debye–Scherrer technique.^43^

**Fig. 2:**
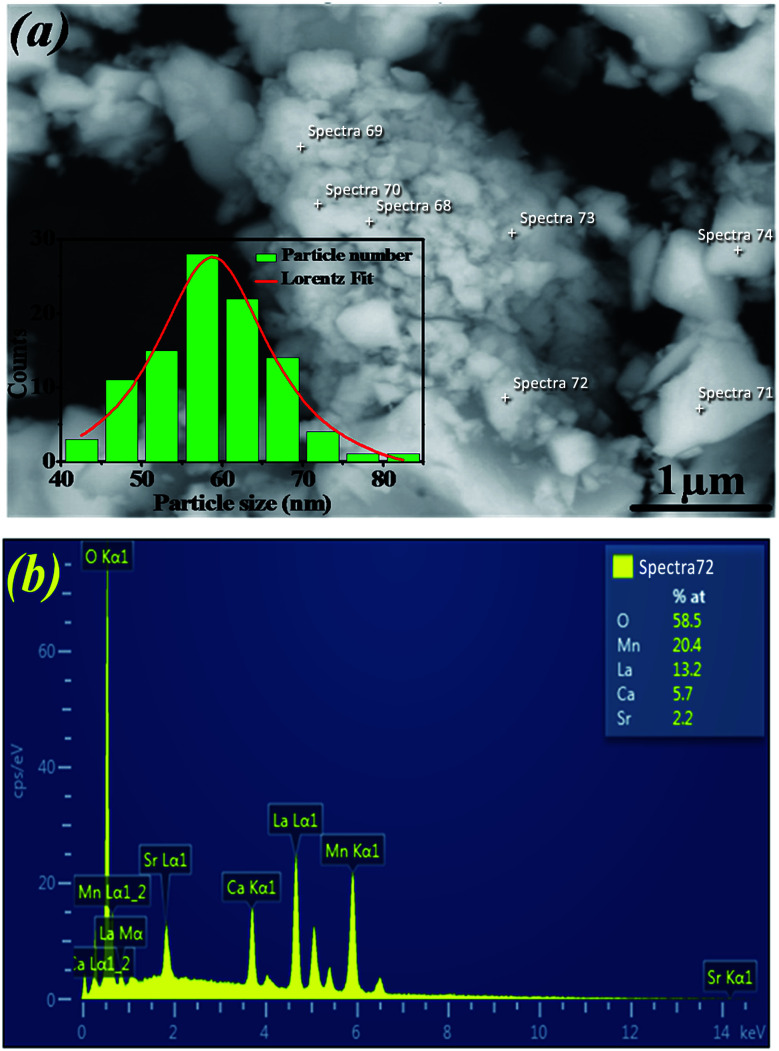
(a) SEM image of La_0.6_Ca_0.3_Sr_0.1_MnO_3_ compound. The inset shows the size distribution histogram. (b) EDAX analysis spectrum.

Fig. 2a shows the SEM micrograph of our synthesized sample. The particles are largely agglomerated with a broad size distribution. The size distribution of particles presented in the inset of Fig. 2a is analyzed quantitatively by fitting the histogram using a Lorentzian function. The mean diameter of La_0.6_Ca_0.3_Sr_0.1_MnO_3_ is mostly 59 nm. The particle size obtained by SEM image is larger than that calculated by XRD data which indicates that each particle observed by SEM is formed by several crystallized grains.

Fig. 2b exemplifies the EDAX spectrum of La_0.6_Ca_0.3_Sr_0.1_MnO_3_ compound. The analysis was carried out on different zones. One can see that there are no impurities. All the elements integrated during the preparation (La, Ca, Sr, Mn and O) are present. The sample composition is similar to the desired one.

### 3.2. Magnetic measurements

**Fig. 3:**
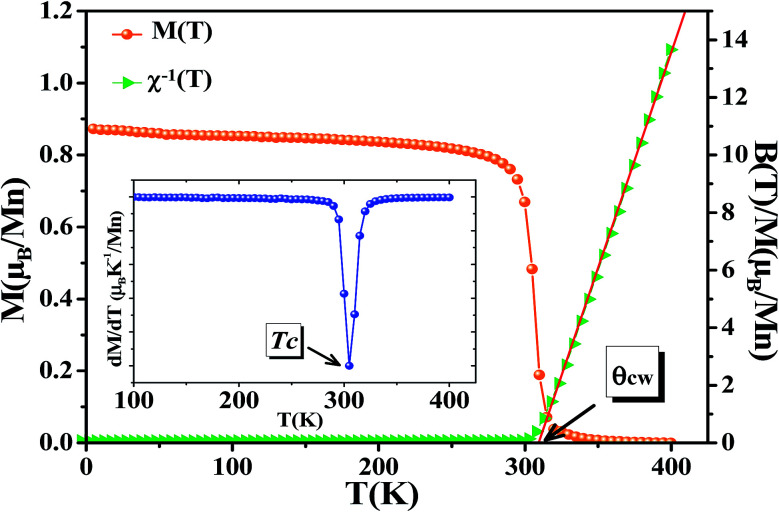
Temperature dependence of magnetization *M*(*T*) and temperature dependence of the inverse magnetic susceptibility *χ*^−1^(*T*) at *μ*_0_*H* = 0.05 T for La_0.6_Ca_0.3_Sr_0.1_MnO_3_ compound (the solid line is the linear fit to the susceptibility data according to Curie–Weiss law above Tc). The inset presents the plot of d*M*/d*T*^−1^ as a function of temperature.

The temperature dependence of magnetization curve is carried out under an applied magnetic field of 0.05 T (Fig. 3). With decreasing temperature, La_0.6_Ca_0.3_Sr_0.1_MnO_3_ exhibits a single magnetic transition from PM to FM phase at Curie temperature (*T*_c_ = 304 K) defined as the temperature at which d*M*/d*T* shows a minimum (inset Fig. 3). Curie temperature near room temperature has a great importance in terms of the cooling technology.^44^

In order to better understand the magnetic behavior of our sample in the PM region above *T*_c_, we studied the inverse magnetic susceptibility as a function of temperature *χ*^−1^(*T*). Fig. 3 shows that *χ*^−1^(*T*) follows the Curie–Weiss law defined as:^45^4
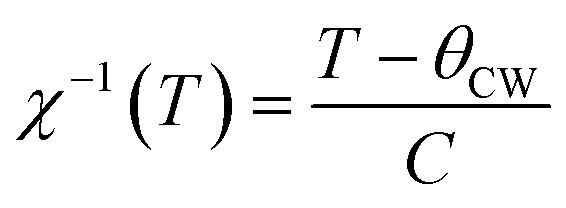
where *θ*_cw_ is Curie–Weiss temperature and *C* is Curie constant.

It is known that the fitting of *χ*^−1^(*T*) curve using Curie–Weiss law provides a valuable information about the magnetic character of material.^46–49^ In our case, by fitting the high temperature region of *χ*^−1^(*T*), the Curie–Weiss temperature *θ*_cw_ proves to be equal to 310 K. The obtained value of *θ*_cw_ is positive, validating the FM character of our sample. Generally, *θ*_cw_ is slightly higher than *T*_c_ which refers basically to the presence of a magnetic inhomogeneity.^50^

The experimental effective paramagnetic moment *μ*^exp^_eff_ can be estimated from the Curie constant by the relation:^51^5
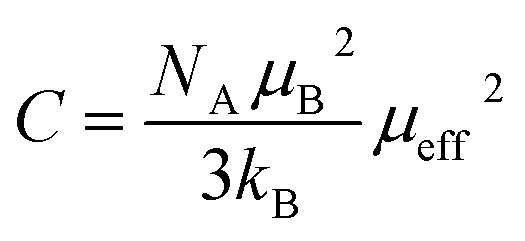
where *N*_A_ is the Avogadro number, *μ*_B_ is the Bohr magneton and *k*_B_ is the Boltzmann constant.

In this paper, the magnetization is expressed in *μ*_B_/Mn. The Curie constant is thus reduced to:6
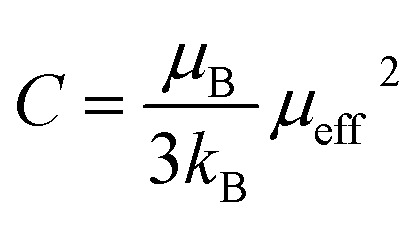


The calculated effective paramagnetic moment *μ*^cal^_eff_ is calculated as follows:^52^7
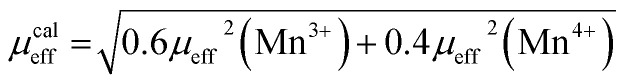
where *μ*_eff_(Mn^3+^) = 4.9 *μ*_B_ and *μ*_eff_(Mn^4+^) = 3.87*μ*_B_.^53^

The obtained values of *μ*^exp^_eff_ and *μ*^cal^_eff_ are found to be equal to 5.57*μ*_B_ and 4.51*μ*_B_, respectively. The difference between the experimental effective paramagnetic moment and the calculated one can be explained by the existence of FM clusters within the PM phase.^54^

**Fig. 4:**
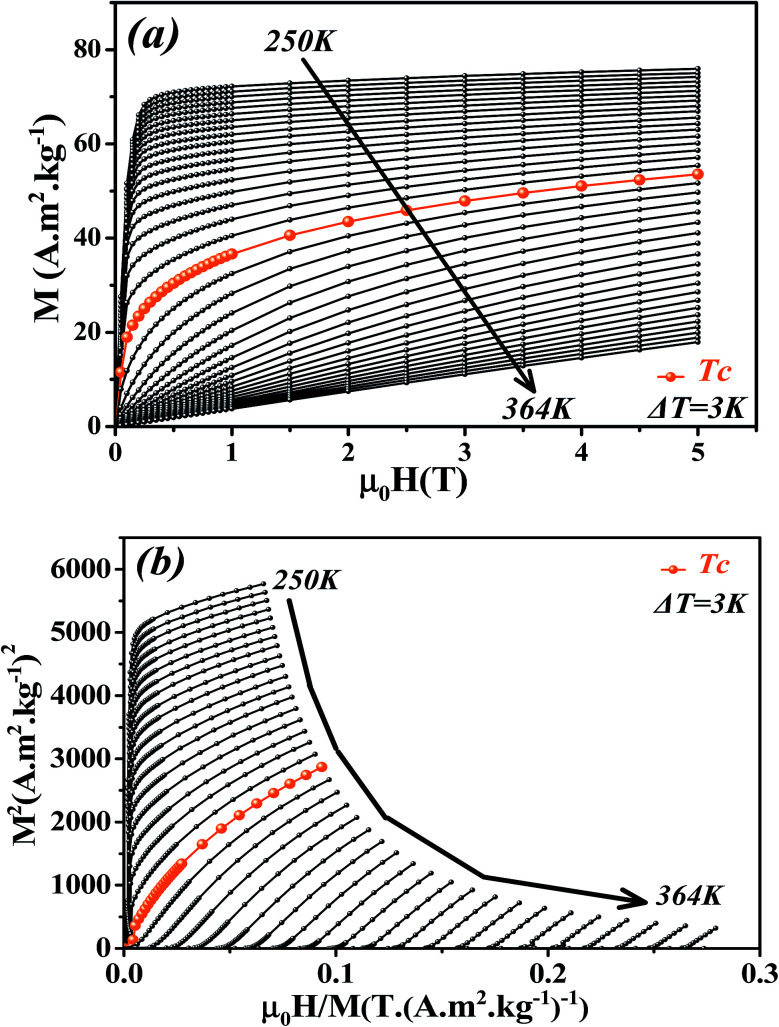
(a) Isothermal magnetization curves measured at different temperatures around *T*_c_ for La_0.6_Ca_0.3_Sr_0.1_MnO_3_ compound. (b) Arrott plots (*M*^2^*vs. μ*_0_*H*/*M*).

The isothermal magnetizations *versus* applied magnetic field *M*(*μ*_0_*H*,*T*) measured at various temperatures with a maximum magnetic field of 5 T are depicted in Fig. 4a. Below *T*_c_, *M*(*μ*_0_*H*,*T*) data increases sharply at low fields and then shows a tendency to saturation as field value increases which is typical for FM materials. Above *T*_c_, a dramatic decrease of *M*(*μ*_0_*H*,*T*) is observed with an almost linear behavior as a feature of PM materials.

Fig. 4b presents the Arrott plots of (*M*^2^*vs. μ*_0_*H*/*M*) which are derived from the isothermal magnetizations. According to the criterion proposed by Banerjee,^55^ the order of the magnetic phase transition can be checked through the sign of the slope of Arrott curves (*M*^2^*vs. μ*_0_*H*/*M*). The positive slope observed for all studied temperatures indicates that the magnetic transition between the FM and PM phase is of the second order.

### 3.3. Magnetocaloric properties

In order to enquire about the efficiency of our compound in the magnetic refrigeration systems, the magnetic entropy change (Δ*S*_M_) can be determined indirectly from the isothermal magnetization curves using the approximated Maxwell equation:^56^8
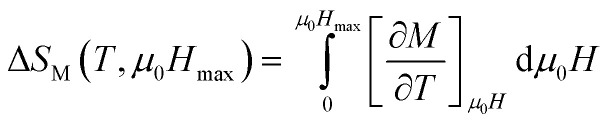


**Fig. 5:**
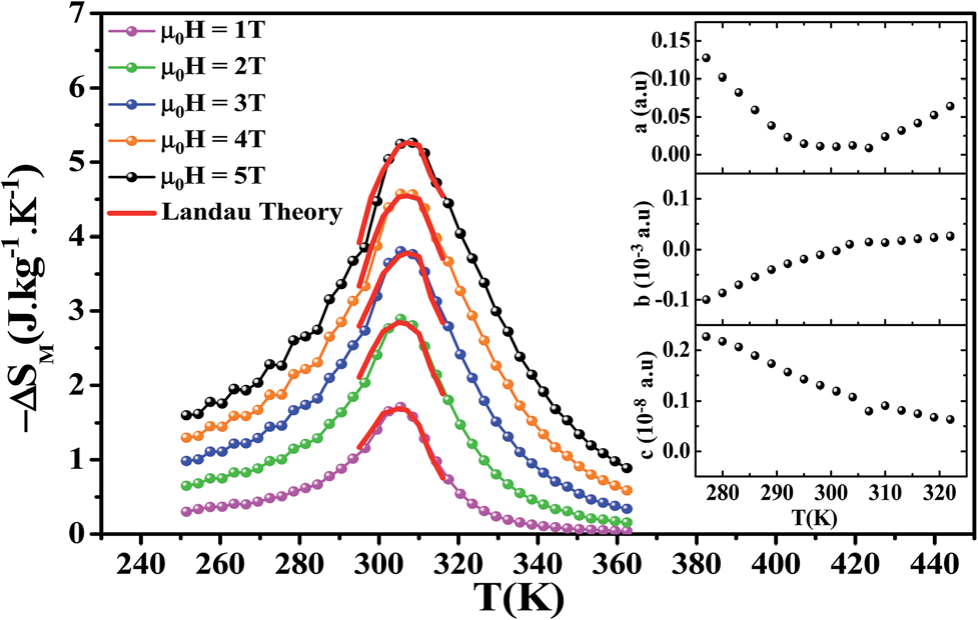
Experimental and theoretical magnetic entropy changes for La_0.6_Ca_0.3_Sr_0.1_MnO_3_ compound under applied fields ranging from 1 to 5 T. The inset displays the temperature dependence of Landau's coefficients.

Fig. 5 shows the temperature dependence of the magnetic entropy change (−Δ*S*_M_(*T*)) under several external magnetic fields for La_0.6_Ca_0.3_Sr_0.1_MnO_3_ sample. The magnitude of Δ*S*_M_ increases with the increase of the applied magnetic field and reaches its maximum around the Curie temperature *T*_c_. The maximum values of the magnetic entropy (−Δ*S*^max^_M_) are 2.89 and 5.26 J kg^−1^ K^−1^ under an applied magnetic field of 2 and 5 T, respectively. These values correspond to about 53 and 52% of those observed in pure Gd at 2 and 5 T, respectively.^5,57,58^

Depending on the magnitude of (−Δ*S*_M_) and its full-width at half maximum (*δT*_FWHM_), the magnetocaloric efficiency can be determined through the relative cooling power (RCP).^59^ The latter, defined as the heat transfer between the hot and the cold sinks in one ideal refrigeration cycle, can be described by the following formula:

9 RCP = (−Δ*S*^max^_M_) × *δT*_FWHM_

The calculated RCP is 98.17 J kg^−1^ for *μ*_0_*H* = 2 T and 262.53 J kg^−1^ for *μ*_0_*H* = 5 T, which stands for about 60 and 64% of that observed in pure Gd, respectively. (−Δ*S*^max^_M_) and RCP constitute a good initial approximation to the potential performance of a material used as a magnetic refrigerator. To evaluate the applicability of our compound as magnetic refrigerant, the obtained values of (−Δ*S*^max^_M_) and RCP in our study, compared to other magnetic materials,^5,58,60–66^ are summarized in Table 2.

**Table 2:** Summary of magnetocaloric properties of La_0.6_Ca_0.3_Sr_0.1_MnO_3_ compound compared to other magnetic materials

Compound	*μ*_0_ *H* (T)	*T*_c_ (K)	(−Δ*S*^max^_M_), (J kg^−1^ K^−1^)	RCP (J kg^−1^)	Ref.
La_0.6_Ca_0.3_Sr_0.1_MnO_3_	5	304	5.26	262.53	Present work
	2		2.89	98.17	
Gd	5	294	10.2	410	5
	2		5.5	164	58
La_0.8_K_0.2_MnO_3_	5	281	3.71	160	60
La_0.67_Ba_0.33_MnO_3_	5	292	1.48	161	61
La_0.7_(Ba, Sr)_0.3_MnO_3_	2	316	1.27	75.74	62
La_0.8_Na_0.2_MnO_3_	2	335	2.83	76.91	63
La_0.75_Sr_0.25_Mn_0.8_Cr_0.2_O_3_	5	278	3.85	323	64
La_0.75_Sr_0.25_Mn_0.95_Ti_0.05_O_3_	2	308	2.2	90	65
La_0.7_Sr_0.3_Mn_0.95_Co_0.05_O_3_	1.5	300	1.17	46.8	66

For the theoretical modeling of the MCE, Amaral *et al.*^29^ attempted to explore in depth the MCE in terms of Landau theory of phase transition which takes into account the electron interaction and magnetoelastic coupling effects.

According to Landau theory, Gibb's free energy is expressed as:^67^10
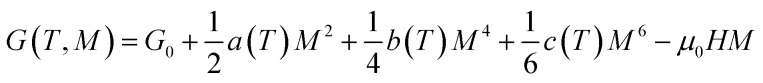
where *a*(*T*), *b*(*T*) and *c*(*T*) are Landau coefficients. These coefficients are temperature-dependent parameters containing the electron condensation energy, the elastic and the magnetoelastic coupling terms of the free energy.^29,68^

Using the equilibrium condition at *T*_c_ (∂*G*/∂*M* = 0), the obtained relation between the magnetization of the material and the applied field is expressed as follows:11
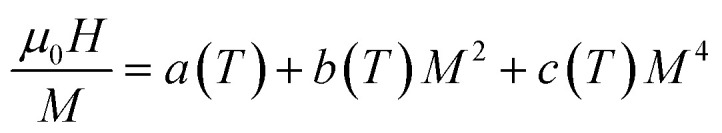


Landau's parameters *a*(*T*), *b*(*T*) and *c*(*T*) determined from a polynomial fit of the experimental isothermal magnetizations are shown in the inset of Fig. 5.

The magnetic entropy change is theoretically obtained from the differentiation of the free energy with respect to temperature as follows:^69^12
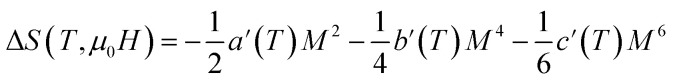
where *a′*(*T*), *b′*(*T*) and *c′*(*T*) are the temperature derivatives of the landau coefficients.

Fig. 5 shows the magnetic entropy behavior of our sample, obtained by comparing the results coming from the Maxwell relation integration of the experimental data and the one calculated by using the Landau theory. An excellent concordance is found between the experimental magnetic entropy change and the theoretical one in the vicinity of the magnetic phase transition. The result indicates that both magnetoelastic coupling and electron interaction can account for the MCE properties of this sample.^70^

From physical point of view, the efficiency of magnetic refrigerant materials can be assessed by the nature of the phase transition that they undergo.^71^ The phase transition can be of the first order in which the first derivative of the Gibb's free energy is discontinuous. Therefore, magnetization shows an abrupt change at the transition temperature. Although this change causes a correspondingly giant magnetic entropy change, this appears at the cost of thermal and magnetic hysteresis, which should be avoided in refrigerators appliances. However, if the magnetic phase transition is of the second order, no thermal and magnetic hysteresis are observed which is much more suitable for refrigerators applications.

To further investigate the nature of the phase transition in samples, Bonilla *et al.*^72^ have suggested a phenomenological universal curve. The construction of the phenomenological universal curve is based on the collapse of all Δ*S*_M_(*T*,*μ*_0_*H*) data measured at different *μ*_0_*H* into one single new curve. This procedure was performed by normalizing the magnetic entropy change curves Δ*S*_M_ with respect to their peak Δ*S*^max^_M_ (Δ*S*_M_/Δ*S*^max^_M_) and rescaling the temperature axis using two additional reference temperatures in a different way below and above *T*_c_. The positions of these additional reference temperatures in the curve correspond to *θ* = ±1:13
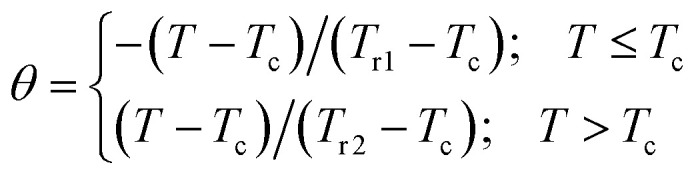
where *θ* is the rescaled temperature, *T*_r1_ and *T*_r2_ are the temperature values of the two reference points of each curve. For the present work, *T*_r1_ and *T*_r2_ have been selected as temperatures corresponding to Δ*S*_M_(*T*_r1,2_) = (1/2)Δ*S*^max^_M_.

**Fig. 6:**
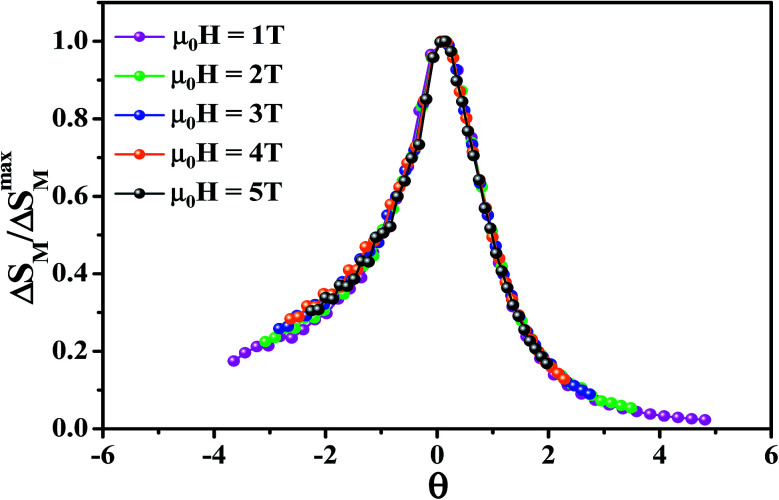
Universal curve of La_0.6_Ca_0.3_Sr_0.1_MnO_3_ compound.

Departing from Fig. 6, it is obvious that all normalized entropy change curves collapse into a single curve confirming that the PM/FM transition observed in our sample is of the second order, which is in good agreement with the analysis of the Banerjee criterion.

### 3.4. Critical behavior determination through magnetic entropy change

Generally, the common methods to identify the critical behavior of materials undergoing second order phase transition are the modified Arrott plots^73^ and the Kouvel–Fisher method.^74^ The choice of model to first construct some tentative Arrott plots and determine initial values of the critical exponents affects systematically their final values. Since several researchers make different choices, a considerable uncertainty is unavoidable. To eliminate the drawbacks arising from the conventional method,^75^ another method based on the field dependence of magnetic entropy change can be used to show the intrinsic relation between MCE and the universality class. According to the approach suggested by Oesterreicher and Paker,^76^ the field dependence of the magnetic entropy change of second order phase transition magnetic materials can be approximated by a universal law of the field:

14 Δ*S*_M_ ∝ (*μ*_0_*H*)^*n*^

The exponent *n* which is dependent on *μ*_0_*H* and *T*, can be calculated as follows:15
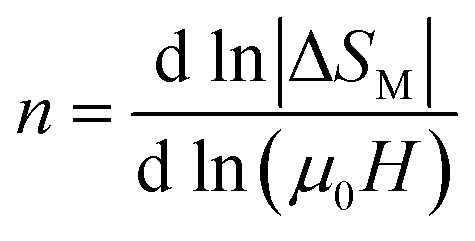


At *T* = *T*_c_, the exponent *n* becomes an independent field:^77^16
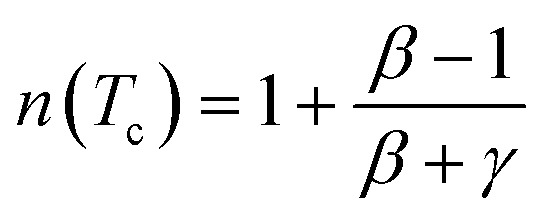
where *β* and *γ* are the critical exponents.

Using *βδ* = *β* + *γ*^78^ the relation (16) can be rewritten as:17
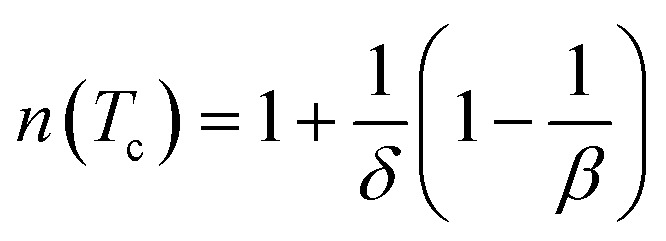


**Fig. 7:**
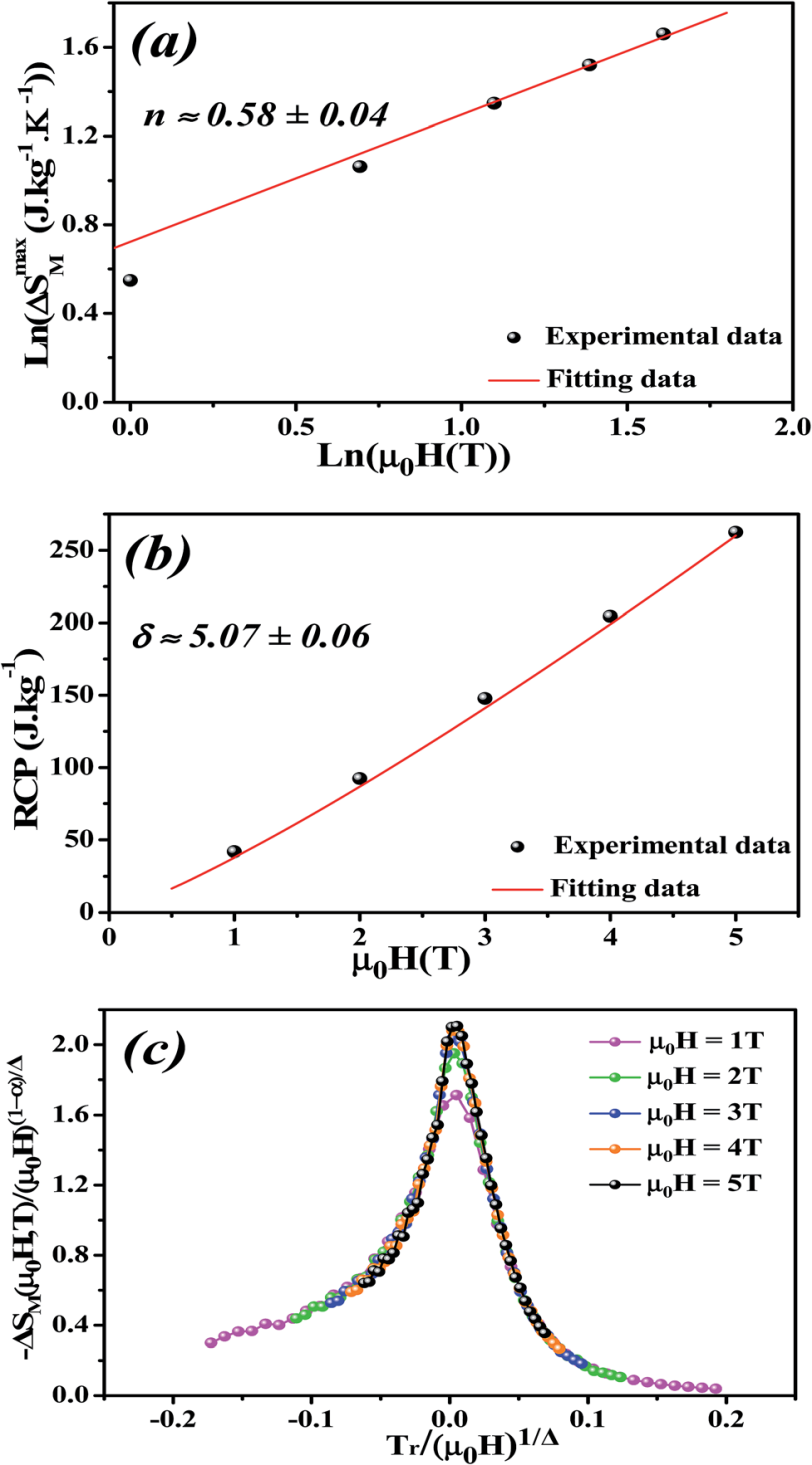
(a) Variation of (ln(Δ*S*^max^_M_) *vs.* ln(*μ*_0_*H*)). (b) Variation of (RCP *vs. μ*_0_*H*). (c) Scaled magnetic entropy change *versus* scaled temperature using critical exponents for La_0.6_Ca_0.3_Sr_0.1_MnO_3_ compound.

By fitting Δ*S*_M_*vs. μ*_0_*H* data on the ln–ln scale (Fig. 7a), the value of *n* obtained from the slope around *T*_c_ is 0.58 ± 0.04. On the basis of the mean-field approach, the field dependence of the magnetic entropy change at the Curie temperature corresponds to *n* = 2/3.^79,80^ The deviation of *n* value from the mean-field behavior refers basically to the presence of magnetic inhomogeneities in the vicinity of transition temperature.^81^

The field dependence of RCP for our sample can be expressed as a power law:^64^18
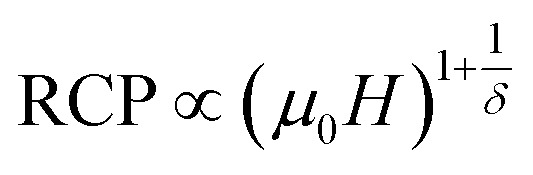
where *δ* is the critical exponent of the magnetic transition.

The value of *δ* obtained from the fitting of RCP *vs. μ*_0_*H* plot is 5.07 ± 0.06 (Fig. 7b). By combining the value of *n* and *δ* according to eqn (16) and (17), the obtained values of the critical exponents *β* and *γ* are 0.319 ± 0.026 and 1.302 ± 0.010, respectively. It is noticed that the values of the critical exponents calculated using the magnetic entropy change match reasonably well within the 3D-Ising model (*β* = 0.325, *γ* = 1.241, *δ* = 4.82).

To check the reliability of the obtained critical exponents, Franco *et al.*^71^ used the scaled equation of state which is expressed as:19
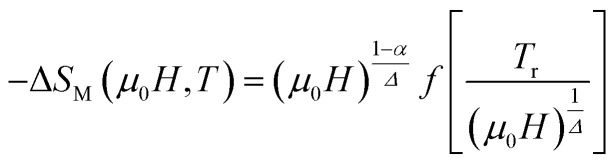
where *α* = 2 − 2*β* − *γ* and *Δ* = *β* + *γ* are the usual critical exponents^82^ and 
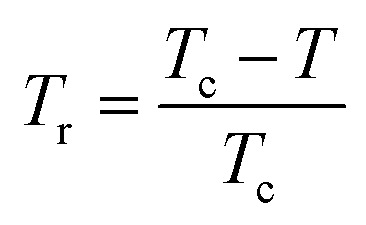
 is the reduced temperature.

According to eqn (19) and using the appropriate values for the critical exponents, the plot of 
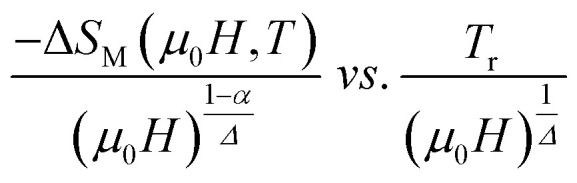
 is depicted in Fig. 7c. All the experimental data clearly collapses on a single master curve for all measured fields and temperatures indicating that the obtained values of the critical exponents for this specimen are in excellent accordance with the scaling hypothesis, which further reinforces their reliability. This result confirms that the critical behavior is well correlated with the MCE properties.

### 3.5. Spontaneous magnetization determination through magnetic entropy change

In the following section, the mean-field theory is invested so as to investigate the spontaneous magnetization (*M*_spont_) in our sample. A general result issued from a mean-field theory reveals that the magnetic entropy as a function of magnetization can be described as:^16,83,84^20
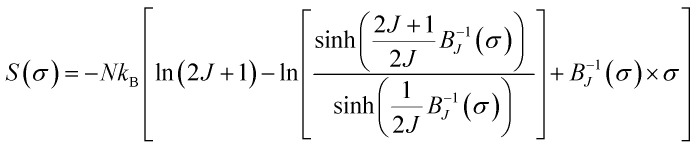
where *N* is the number of spins, *k*_B_ is the Boltzmann constant, *J* is the spin value, *B*_*J*_ is the Brillouin function for a given *J* value and *σ* = *M*/*NJgμ*_B_ is the reduced magnetization.

For small *M* values, a proportionality of magnetic entropy to *σ*^2^ can be defined as:21
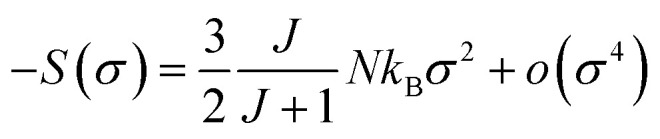


In the FM state, the system presents a spontaneous magnetization, therefore *σ* ≠ 0. Consequently, considering only the first term of eqn (21), the magnetic entropy may be written as:22
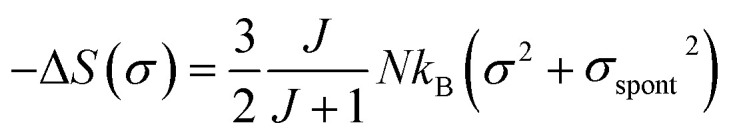


Taking the square of the reduced magnetization and substituting it for *σ*^2^ in eqn (22) results in:23
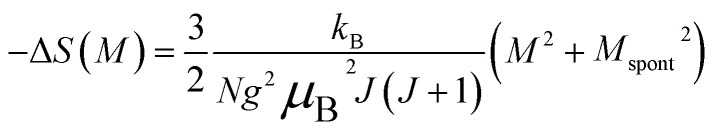
where *g* is the gyromagnetic ratio.

**Fig. 8:**
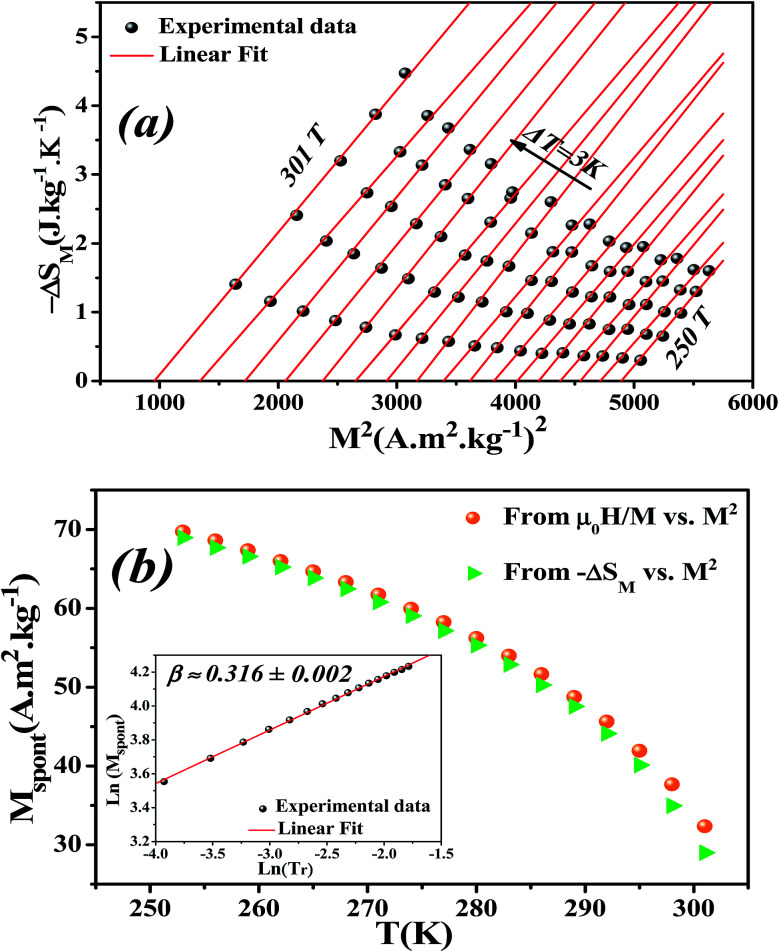
(a) Isothermal (−Δ*S*_M_*vs. M*^2^)curves. (b) Spontaneous magnetization of La_0.6_Ca_0.3_Sr_0.1_MnO_3_ compound, deduced from the extrapolation of the isothermal (−Δ*S*_M_*vs. M*^2^) curves and from the Arrott plots (*M*^2^*vs. μ*_0_*H*/*M*). The inset shows (ln(*M*_spont_) *vs.* ln(*T*_r_)).

Eqn (23) implies that in the FM region, the isothermals (−Δ*S*_M_) *vs. M*^2^ exhibit a linear variation. By fitting the (−Δ*S*_M_) *vs. M*^2^ curves for *T* < *T*_c_, the value of *M*_spont_ can be estimated through the intersection of the straight lines with the *M*^2^ axis (Fig. 8a). For *T* > *T*_c_, the (−Δ*S*_M_) *vs. M*^2^ plots start at a null *M* value.

The spontaneous magnetization *M*_spont_ as a function of temperature is plotted in Fig. 8b. As the temperature decreases, the spontaneous magnetization increases, suggesting that the system is approaching a spin ordering state at lower temperature. The values of *M*_spont_, estimated from the analysis of the magnetization dependence of magnetic entropy change (Δ*S*_M_*vs. M*^2^), are compared with those deduced from the classical extrapolation of the Arrott curves (*μ*_0_*H*/*M vs. M*^2^), as shown in Fig. 8b. The excellent agreement between both methods confirms the validity of the method based on the magnetic entropy change to determine the spontaneous magnetization of the La_0.6_Ca_0.3_Sr_0.1_MnO_3_ system as well as that of other compounds.

It is known that the spontaneous magnetization near Curie temperature of a second order phase transition material corresponds to a critical exponent *β*, through the relation:^85^

24 *M*_spont_ ∝ (*T*_r_)^*β*^

with 
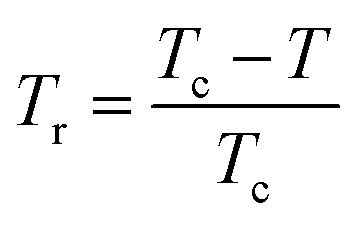
 is the reduced temperature.

By changing eqn (24) to ln–ln scale, the value of *β* corresponds to the slope of the curve. From the linear fitting shown in the inset of Fig. 8b, we have obtained *β* = 0.316 ± 0.002, which is consistent with the 3D-Ising model (*β* = 0.325), as mentioned in the previous section.

## 4. Conclusion

In summary, a detailed study of magnetocaloric properties of La_0.6_Ca_0.3_Sr_0.1_MnO_3_ compound has been systematically performed. Through thermodynamic Maxwell relations, the magnetic entropy change (Δ*S*_M_) has been determined. Our compound presents large magnetocaloric effect (MCE) values around room temperature. It exhibits a relative cooling power (RCP) corresponding to about 64% of that observed in pure Gd for *μ*_0_*H* = 5 T, indicating its potential application in the cooling fields. The analysis of (Δ*S*_M_) using Landau theory is consistent with that estimated by Maxwell relations, concluding the importance of magnetoelastic coupling and electron interaction in the MCE properties of manganites system. Banerjee criterion and a phenomenological universal curve of the magnetic entropy change have successfully confirmed the second order of the magnetic phase transition. The field dependence of the magnetic entropy change was applied to study the critical behavior. Our results go in tandem with the values corresponding to the 3D-Ising model. The obtained critical exponents follow the scaling laws which further confirm their reliability. The field dependence of magnetic entropy change can be effectively used in studying the critical behavior of magnetic materials. The methodology based on the analysis of the magnetic entropy change (Δ*S*_M_*vs. M*^2^) compared to the classical extrapolation of the Arrott curves (*μ*_0_*H*/*M vs. M*^2^) proves that magnetic entropy change is a valid approach to determine the spontaneous magnetization in La_0.6_Ca_0.3_Sr_0.1_MnO_3_ system.

## Conflicts of interest

There are no conflicts to declare.

## Supplementary Material
